# Mental Health Variables Impact Weight Loss, Especially in Patients with Obesity and Binge Eating: A Mediation Model on the Role of Eating Disorder Pathology

**DOI:** 10.3390/nu15183915

**Published:** 2023-09-09

**Authors:** Jacopo Pruccoli, Isabelle Mack, Bea Klos, Sandra Schild, Andreas Stengel, Stephan Zipfel, Katrin Elisabeth Giel, Kathrin Schag

**Affiliations:** 1 Pediatric Neurology and Psychiatry Unit, IRCCS Istituto delle Scienze Neurologiche di Bologna, Regional Center for Feeding and Eating Disorders in the Developmental Age, 40138 Bologna, Italy; jacopo.pruccoli2@unibo.it; 2 Department of Medical and Surgical Sciences, University of Bologna, 40126 Bologna, Italy; 3 Department of Psychosomatic Medicine and Psychotherapy, Medical University Hospital Tübingen, 72076 Tübingen, Germany; bea.klos@med.uni-tuebingen.de (B.K.); sandra.schild@med.uni-tuebingen.de (S.S.); andreas.stengel@med.uni-tuebingen.de (A.S.); stephan.zipfel@med.uni-tuebingen.de (S.Z.); katrin.giel@med.uni-tuebingen.de (K.E.G.); kathrin.schag@med.uni-tuebingen.de (K.S.); 4 Centre of Excellence for Eating Disorders Tübingen (KOMET), 72076 Tübingen, Germany; 5 DZPG (German Center for Mental Health), 72076 Tübingen, Germany; 6 Department for Psychosomatic Medicine, Charité Center for Internal Medicine and Dermatology, Charité-Universitätsmedizin Berlin, Corporate Member of Freie Universität Berlin, Humboldt-Universität at Berlin, and Berlin Institute of Health, 12203 Berlin, Germany

**Keywords:** binge eating, behavior weight loss intervention, eating behavior, impulsivity, mental health, obesity

## Abstract

Background: Various mental health and eating behavior variables have been independently associated with predicting weight loss in individuals with obesity. This study aims to investigate a mediation model that assesses the distinct contributions of these variables in predicting weight changes in patients with obesity following an outpatient behavioral weight loss intervention (BWLI). Methods: General mental health (depression, anxiety, stress, impulsivity), eating behavior (cognitive restraint, disinhibition, hunger), eating disorder pathology, and body mass index (BMI) were assessed in a group of 297 patients with obesity at the admission of a BWLI program. BMI was re-evaluated during the final treatment session. A mediation model was employed to examine whether mental health and eating behavior variables predicted BMI changes, with eating disorder pathology serving as a mediator. The model was tested both overall and within two patient subgroups: those with regular binge eating (≥four episodes/month) and those without. Results: In the overall sample (n = 238), the relationships between depression, impulsivity, and cognitive restraint with BMI change were mediated by eating disorder pathology. In the subgroup with regular binge eating (n = 99, 41.6%), the associations between stress and disinhibition with BMI change were additionally mediated by eating disorder pathology. In the subgroup without regular binge eating, eating disorder pathology showed no mediating effect. Discussion: Multiple mental health and eating behavior variables assessed at admission predicted BMI changes, particularly when mediated by eating disorder pathology in patients with regular binge eating. A comprehensive psychopathological assessment prior to starting BWLI may help identify multiple factors affecting prognosis and treatment outcomes. Long-term follow-up studies in this field are required.

## 1. Introduction

According to the World Health Organization, obesity has become a worldwide issue, with rates having tripled since 1975 [1], and it is recognized as a major risk factor for chronic diseases, responsible for 74% of all deaths [2]. Additionally, obesity significantly affects mental health and overall quality of life [3]. The core focus of obesity research lies in identifying potential etiological factors, prevention strategies, and intervention programs [3,4]. Apart from somatic and sociodemographic risk factors, behavioral factors such as physical inactivity and malnutrition have been involved in the pathogenesis of obesity [4]. Treatments for obesity primarily aim to reduce energy intake and body weight and may include conservative weight-loss interventions, bariatric surgery, and/or adjuvant pharmacotherapy [5]. According to most guidelines addressing obesity, the initial approach involves conservative weight-loss interventions that target comprehensive lifestyle modifications, including dietary habits and physical activity [6,7]. These treatments have reported average weight losses of 5% to 7% [5], with consistent outcomes observed across different obesity categories [8]. Mental health and behavioral factors play a role in predicting the success of weight-loss programs and may impact the maintenance of positive outcomes [9].

**Figure 1:**
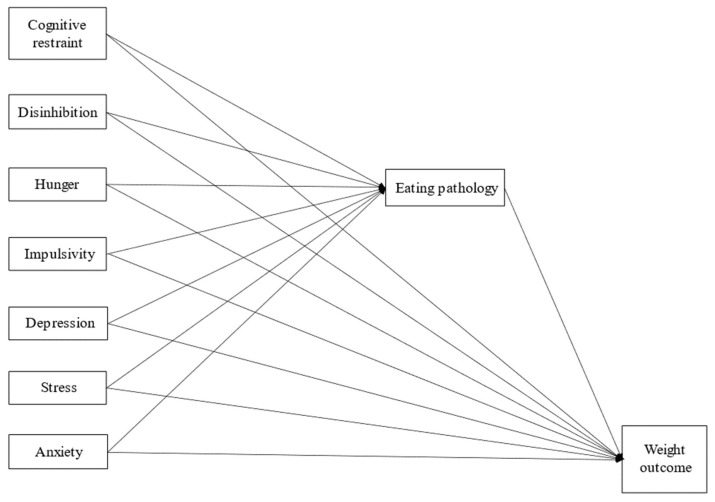
Theoretical model proposing eating disorder pathology as a mediator between weight change after an outpatient BWLI and several mental health- and eating behavior-related variables.

Elevated rates of comorbid depression, anxiety, and stress have been documented in individuals with obesity, particularly when Metabolic Syndrome co-occurs [3]. Furthermore, depressive symptoms, anxiety, and stress may lead to increased and dysfunctional eating behaviors. Previous research in patients with obesity has established the predictive roles of impulsivity [10], depression [11], stress [12], and anxiety [13], as well as the different components of eating behavior, including cognitive restraint [14], disinhibition [14,15], and hunger [16]. Additionally, a relationship between these variables and eating disorder pathology has been consistently reported across multiple studies. Impulsivity [17,18], depression [19,20], anxiety [21], stress [22], cognitive restraint [23], disinhibition [19], and hunger [24] have all been associated with eating disorder pathology. Consequently, we propose a theoretical model in which eating disorder pathology may act as a mediator between these variables and weight change in patients with obesity (see Figure 1). Despite the existing background, no single study has yet investigated the potential contributions of these distinct variables within a unified theoretical model.

Furthermore, a specific subgroup of patients with obesity, namely those with Binge Eating Disorder (BED), appears to be particularly affected by psychological factors. They exhibit increased levels of impulsivity, anxiety, and depression in comparison to patients with obesity who do not engage in binge eating. Moreover, patients with obesity who do and do not engage in binge eating display distinct neurobiological characteristics [25,26,27,28,29]. BED stands out as the most prevalent among specific eating disorders [30]. It is characterized by regular binge eating episodes, involving the consumption of a large quantity of food until feeling uncomfortably full, accompanied by a pervasive sense of loss of control [31]. This loss of control represents the main criterion for BED and is emphasized in the ICD-11 diagnostic criteria [32]. Notably, the experience of loss of control closely parallels the characteristics of impulsivity, manifesting as impulsive and spontaneous eating without foresight and difficulties in halting or inhibiting eating behaviors [33,34].

Building upon existing evidence, the present study aims to investigate a potential theoretical model (Figure 1) designed to predict weight change in individuals with obesity after a conservative outpatient behavioral weight-loss intervention (BWLI) [35]. Our study relies on the results of a previous investigation conducted by Mack et al. [36] and the model proposed by Schag et al. [37]. Schag’s model posits that the relationship between weight loss and general mental health variables, such as impulsivity and depression, is mediated by eating disorder pathology [37]. Consistent with these studies and the background provided, we hypothesize that impulsivity, depression, stress, anxiety, and eating behavior may be associated with weight loss through the mediation of eating disorder pathology. To test this hypothesis, we longitudinally assessed self-reported psychopathological and eating-behavior variables at admission to a BWLI, and we measured weight changes upon discharge. We further hypothesize that a specific subgroup of patients with obesity, characterized by regular binge eating, may show even stronger associations with the investigated mental and eating behavior risk factors. Consequently, we anticipate that eating disorder pathology may represent a stronger mediator between these variables and weight change within this subgroup.

## 2. Materials and Methods

### 2.1. Study Design, Participants, and Procedures

We report data from a prospective study, conducted on patients undergoing the VIADUKT treatment at the University Hospital Tübingen, Germany. The study has been approved by the Ethics Committee of the University Hospital Tübingen, Germany—protocol number 391/2019BO2.

VIADUKT entails a state-of-the-art multimodal BWLI for individual outpatients with obesity. The VIADUKT protocol consists of 10 structured group meetings (focusing on nutritional education and healthy lifestyle promotion) and 20 guided exercise sessions. The included patients received education on motivational approaches, flexible and controlled eating patterns, the fundamentals of regular exercise, stress management skills, and techniques for maintaining weight loss for the long term. The intervention follows German clinical practice guidelines for obesity [38]. The procedures involved in the VIADUKT program are reported in detail elsewhere [35].

For the purpose of the study, we considered data from the time interval spanning May 2014 (the program’s start) to September 2019. Baseline characteristics, such as general psychopathology (impulsivity, anxiety, depression, and stress), eating behavior (restraint, disinhibition, and hunger), as well as eating disorder pathology, were assessed during the first session of the VIADUKT treatment using validated questionnaires. Height and weight, necessary to compute body mass index (BMI), were measured at the first VIADUKT session, and weight was again assessed in the last session.

The inclusion criterion for all patients was an age of 18 years or older. A BMI of at least 30 was preferred but not mandatory as long as the indication for treatment of overweight was required according to the national guidelines. Difficulties with the German language represented an exclusion criterion for participation. The total sample comprised 297 patients, with 238 (80.1%) patients included in the overall analysis due to missing data (for details, refer to Section 2.3). Only 17 (7.1%) patients did not complete the VIADUKT treatment as prescribed. Of the total, 139 (58.4%) patients presented obesity without regular episodes of binge eating, while 99 (41.6%) exhibited regular episodes of binge eating (for additional information, see Section 2.2.6).

### 2.2. Measurements

#### 2.2.1. BMI

BMI at the first and last session of the BWLI was calculated based on body weight and height (kg/m^2^). To assess changes, the difference between BMI at admission and discharge was considered.

#### 2.2.2. Impulsivity

The German short version of the Barratt Impulsiveness Scale (BIS) [39,40] was used to assess impulsivity. The BIS-15 represents a self-report questionnaire composed of 15 items in three subscales, rated on a four-point response format, which ranges from 1 = “rarely/never” to 4 = “almost always”. The total score of the BIS scale was considered in this study to measure general impulsivity. Cronbach’s alpha for the BIS total score was 0.58 in this sample.

#### 2.2.3. Depression

The German version of the Patient Health Questionnaire (PHQ-D) represents a screening tool for mental disorders [17]. For this study, the nine-item module assessing depression (PHQ-9) was considered. This module presents a four-point response format ranging from 0 (“not at all”) to 3 (“nearly every day”), with a severity score (ranging from 0 to 27) including cut-offs for mild (5) and moderate (10) depression. Cronbach’s alpha for the PHQ-9 total score was 0.86 in this sample.

#### 2.2.4. Stress

The Perceived Stress Questionnaire (PSQ) was used to assess perceived stress. The PSQ short version consists of 20 items, assessing the stressful life events and circumstances triggering or exacerbating disease symptoms. Higher scores indicate greater levels of stress. For this study, the total score was considered [41]. Cronbach’s alpha for the PSQ total score was 0.41 in this sample.

#### 2.2.5. Anxiety

The Generalized Anxiety Disorder (GAD-7) questionnaire [42] was adopted to assess anxiety symptoms. The GAD-7 represents a one-dimensional instrument investigating symptoms of generalized anxiety disorder according to the criteria reported in the DSM-IV. Scores for the included items range from 0 (not at all) to 3 (nearly every day), providing a sum score ranging from 0 to 21. Cronbach’s alpha for the GAD-7 total score was 0.90 in this sample.

#### 2.2.6. Eating Disorder Pathology and Frequency of Binge Eating

Eating disorder pathology was assessed using the Eating Disorder Examination Questionnaire (EDE-Q), consisting of four subscales: “restraint scale”, “eating concern”, “weight concern”, and “shape concern” [43]. A total score of this scale, measuring the global relevance of eating disorder pathology, was considered in this study. Question 15 of the EDE-Q questionnaire asks the respondent to report the frequency of episodes of binge eating occurring in the last 28 days [43]. For this study, patients who reported at admission a frequency of four or more episodes of binge eating per 28 days were included in the “obesity with regular binge eating” group, while patients reporting three or fewer episodes were included in the “obesity without regular binge eating” group. Cronbach’s alpha for the EDE-Q total score was 0.81 in this sample.

#### 2.2.7. Eating Behavior

The German version [44] of the three-factor eating questionnaire (TFEQ) [45] was used to assess the different components of eating behavior. This questionnaire consists of three scales, which conceptualize eating behavior by behavioral, cognitive, and affective components. The restraint subscale describes strategic dieting behavior, self-regulation, and avoidance of fattening foods. The disinhibition subscale describes habitual, emotional, and situational susceptibility. The hunger subscale describes internal and external processing for hunger cues [44]. Higher scores indicate a higher relevance of the considered eating behavior. Cronbach’s alpha for the TFEQ total score was 0.71 for this sample.

### 2.3. Statistical Analysis

On the sample of patients included in VIADUKT (n = 297), 53 (17.9%) were excluded due to missing data on the psychopathological assessment. Patients were retained if they presented available data from all the considered questionnaires (BIS, PHQ-9, PSQ, GAD-7, EDE-Q, and TFEQ) for at least one time point (admission or discharge). The patients whose data were not available from one or more of the considered questionnaires at both time points were excluded from this study. Patients with missing data from single items were not excluded; as an exception, those with missing data on EDE-Q Item 15 (assessing binge eating frequency) were excluded from this study. Thus, six (2%) patients were excluded due to missing data on the frequency of binge-eating episodes. Finally, 238 (80.1%) patients were included in the analyses. Missing data for the patients included in the present study were imputed. The analysis and imputation of missing data for the considered psychopathological variables (BIS, PHQ-9, PSQ, GAD-7, EDE-Q, and TFEQ) were conducted with multiple imputations [46], generating five imputed datasets. Descriptive analyses were provided overall (238 patients), as well as for the two subgroups (patients with and without regular binge eating). The data were tested for normal distribution using the Kolmogorov–Smirnov test. Potential differences between the two subgroups in the distribution of demographic (gender and age), anthropometric (admission and discharge BMI, as well as admission–discharge BMI difference), and psychopathology/eating behavior (BIS, PHQ-9, PSQ, GAD-7, EDE-Q, and TFEQ) data were assessed. Chi-square tests were used to assess differences in the distribution of nominal variables, while t-tests (non-parametric where needed) were used to assess differences in the distribution of continuous variables. Bivariate correlation analyses (Person’s r for parametric data and Spearman’s rho for non-parametric data) were conducted on pooled data for admission–discharge BMI differences and psychopathological variables.

We tested a theoretical model aimed at explaining the difference between admission and discharge BMI (Figure 1). In this model, we considered admission total scores for BIS, PHQ-9, PSQ, GAD-7, EDE-Q, and the three subscales of TFEQ as potential predictors, while the EDE-Q score at admission was regarded as a potential mediator (see Figure 1). To examine the theoretical model and determine the extent of explained variance, we conducted regression and mediation analyses. Due to the existing evidence from independent studies demonstrating the relationship among all the psychopathological variables, eating behavior, and weight outcomes in patients with obesity [10,11,12,13,14,15,16,17,18,19,20,21,22,23,24], we assessed the potential mediation model independently of the results of simple bivariate correlations, following the principles outlined by [47].

Descriptive analyses and multiple imputations were performed with SPSS Version 26 for Windows. Mediation analyses were performed using R version 4.1.2 for Windows, adopting the “lavaan” package to run mediation analyses on multiple imputed data [46]. Concerning the mediation analysis, we used a bootstrapping analysis with 1.000 samples and a 95% confidence interval. A *p*-value of <0.05 was considered statistically significant.

## 3. Results

### 3.1. Sample Characteristics

Patients with obesity either with or without regular binge eating differed for a series of psychopathological variables. No significant difference between patients with and without regular binge eating was documented for a previous history of weight loss, as well as the time passed since the maximum weight was recorded and the reported maximum previous weight. Patients with obesity and regular binge eating showed higher total BIS, GAD, PSQ, PHQ-9, EDE-Q, and TFEQ (disinhibition and hunger) scores and higher binge-eating frequency in comparison with patients without regular binge eating, but lower TFEQ cognitive restraint scores. Sample characteristics are presented in Table 1.

### 3.2. Correlation Analyses

We found several correlations between the examined variables (Table 2), especially the EDE-Q total score correlated with all questionnaire scores, as well as with admission–discharge BMI difference.

### 3.3. Mediation Analyses

#### 3.3.1. Overall Sample

**Figure 2:**
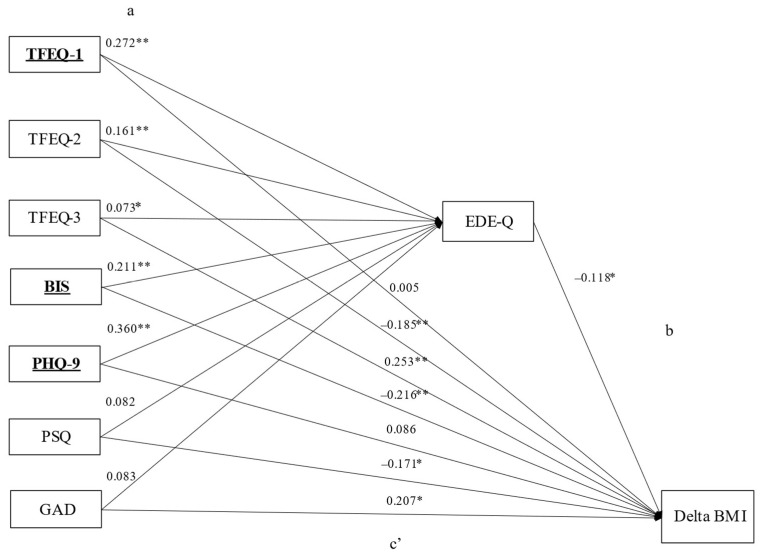
Mediation model for the overall sample. Notes: (**a**) Standardized coefficients for regressions between psychopathology/eating behavior variables and EDE-Q; (**b**): Standardized coefficients for regression between EDE-Q and admission-discharge BMI difference. (**c’**): Standardized coefficients for the direct path between psychopathology/eating behavior variables and admission-discharge BMI difference, controlling for the mediating variable (EDE-Q). The indirect path (**a**,**b**), as well as full data for (**a**,**c’**) paths are reported in Supplementary Material Table S1. * = *p* < 0.05; ** = *p* < 0.001. Independent variables involved in the final mediation model are reported in bold and underlined. Abbreviations: BIS: Barratt Impulsiveness Scale, total score; EDE-Q: Eating Disorder Examination Questionnaire, total score; Delta BMI: body mass index admission-discharge difference; GAD: Generalized Anxiety Disorder Scale, total score; PHQ: Patient Health Questionnaire; PSQ: Perceived Stress Questionnaire, total score; TFEQ-1: Three-Factor-Eating-Questionnaire, cognitive restraint; TFEQ-2: Three-Factor-Eating-Questionnaire, disinhibition, TFEQ-3: Three-Factor-Eating-Questionnaire, hunger.

Figure 2 reports the mediation analysis conducted in the overall sample (patients with obesity, both with and without regular binge eating). The standardized regression coefficients between the independent general psychopathology (PHQ-9, PSQ, GAD, BIS) and eating behavior (TFEQ) variables, and EDE-Q score (Path a) were significant for all the independent variables, except for the PSQ and GAD scores (TFEQ cognitive restraint: *p* < 0.001; TFEQ disinhibition: *p* < 0.001; TFEQ hunger: *p* = 0.046; BIS: *p* < 0.001; PHQ-9: *p* < 0.001), with an explained variance of EDE-Q of R^2^ = 45.8% (*p* < 0.001). The standardized regression coefficient between EDE-Q and admission–discharge BMI difference (Path b) was significant (*p* = 0.025). The bootstrapped indirect effect of the independent general psychopathology and eating behavior variables on admission–discharge BMI difference through the EDE-Q score (path ab) was statistically significant for the scales TFEQ cognitive restraint (*p* = 0.029), BIS (*p* = 0.030), and PHQ-9 (*p* = 0.032). The direct effect between these scales and the admission–discharge BMI difference (path c’) was significant for the BIS scale (*p* < 0.001), TFEQ disinhibition (*p* < 0.001), TFEQ hunger (*p* < 0.001), PSQ (*p* = 0.003), and GAD (*p* = 0.003), but not for the TFEQ cognitive restraint and PHQ-9 scales. Thus, the associations between the TFEQ cognitive restraint, PHQ-9 and BIS scales, and admission–discharge BMI difference were fully (TFEQ cognitive restraint, PHQ-9) or partially (BIS) mediated by the EDE-Q, with an explained variance of admission–discharge BMI difference of R^2^ = 9.8% (*p* < 0.001). Standardized regression coefficients and *p*-values for Paths a, c’ (direct), and ab (indirect) are reported in Supplementary Table S1.

#### 3.3.2. Patients with Obesity and with Regular Binge-Eating Episodes

**Figure 3:**
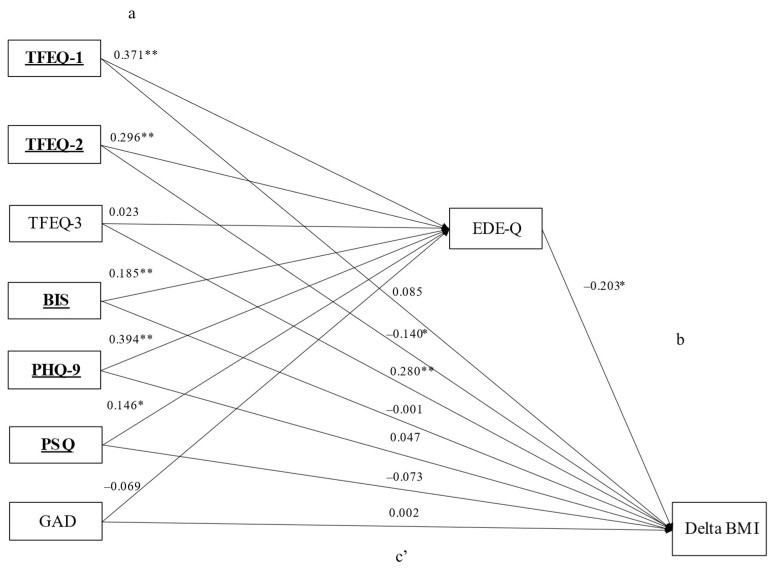
Mediation model for patients with obesity and regular binge eating. Notes: (**a**) Standardized coefficients for regressions between psychopathology/eating behavior variables and EDE-Q; (**b**): Standardized coefficients for regression between EDE-Q and admission-discharge BMI difference. (**c’**): Standardized coefficients for the direct path between psychopathology/eating behavior variables and admission-discharge BMI difference, controlling for the mediating variable (EDE-Q). The indirect path (**a**,**b**), as well as full data for (**a**,**c’**) paths are reported in Supplementary Material Table S2. * = *p* < 0.05; ** = *p* < 0.001. Independent variables involved in the final mediation model are reported in bold and underlined. Abbreviations: BIS: Barratt Impulsiveness Scale, total score; EDE-Q: Eating Disorder Examination Questionnaire, total score; Delta BMI: body mass index admission-discharge difference; GAD: Generalized Anxiety Disorder Scale, total score; PHQ: Patient Health Questionnaire; PSQ: Perceived Stress Questionnaire, total score; TFEQ-1: Three-Factor-Eating-Questionnaire, cognitive restraint; TFEQ-2: Three-Factor-Eating-Questionnaire, disinhibition, TFEQ-3: Three-Factor-Eating-Questionnaire, hunger.

Figure 3 reports the mediation analysis conducted in patients with obesity and with regular binge eating. The standardized regression coefficients between the independent general psychopathology and eating behavior variables and EDE-Q score (path a) were significant for all the independent variables, except for the TFEQ hunger and GAD scores (TFEQ cognitive restraint: *p* < 0.001; TFEQ disinhibition: *p* < 0.001; BIS: *p* < 0.001; PHQ-9: *p* < 0.001; PSQ: *p* = 0.005), with an explained variance of EDE-Q of R^2^ = 51.4% (*p* < 0.001). The standardized regression coefficient between EDE-Q and admission–discharge BMI difference (path b) was significant (*p* = 0.001). The bootstrapped indirect effect of the independent general psychopathology and eating behavior variables on admission–discharge BMI difference through the EDE-Q score (path ab) was statistically significant for all the scales, except for TFEQ hunger and GAD (TFEQ cognitive restraint: *p* = 0.002; TFEQ disinhibition: *p* = 0.002; BIS: *p* = 0.005; PHQ-9: *p* = 0.002; PSQ: *p* = 0.044). The direct effect between these scales and admission–discharge BMI difference (path c’) was significant for the subscale TFEQ disinhibition (*p* = 0.007) and TFEQ hunger (*p* < 0.001), but not for TFEQ cognitive restraint, BIS, PHQ-9, and PSQ. Thus, the associations between the TFEQ cognitive restraint, TFEQ disinhibition, BIS, PHQ-9, and PSQ scales and admission–discharge BMI difference were fully (TFEQ cognitive restraint, BIS, PHQ-9, and PSQ) or partially (TFEQ disinhibition) mediated by the EDE-Q, with an explained variance of admission–discharge BMI difference of R^2^ = 8.0% (*p* < 0.001). Standardized regression coefficients and *p*-values for Paths a, c’ (direct), and ab (indirect) are reported in Supplementary Table S2.

#### 3.3.3. Patients with Obesity and without Regular Binge Eating Episodes

**Figure 4:**
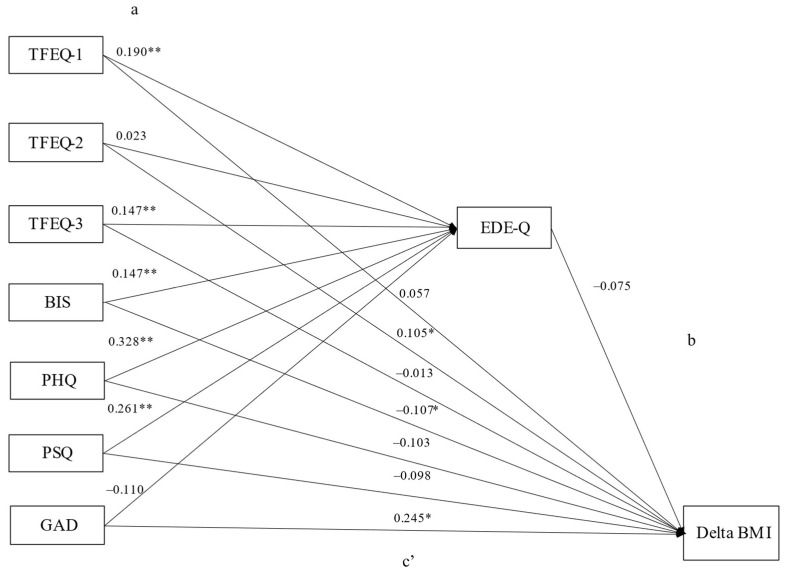
Mediation model for patients with obesity without regular binge eating. Notes: (**a**) Standardized coefficients for regressions between psychopathology/eating behavior variables and EDE-Q; (**b**): Standardized coefficients for regressions between EDE-Q and admission–discharge BMI difference. (**c’**): Standardized coefficients for the direct path between psychopathology/eating behavior variables and admission–discharge BMI difference, controlling for the mediating variable (EDE-Q). The indirect path (**a**,**b**), as well as full data for (**a**,**c’**) paths, are reported in Supplementary Material Table S3. * = *p* < 0.05; ** = *p* < 0.001. Abbreviations: BIS: Barratt Impulsiveness Scale, total score; EDE-Q: Eating Disorder Examination Questionnaire, total score; Delta BMI: body mass index admission-discharge difference; GAD: Generalized Anxiety Disorder Scale, total score; PHQ: Patient Health Questionnaire; PSQ: Perceived Stress Questionnaire, total score; TFEQ-1: Three-Factor-Eating-Questionnaire, cognitive restraint; TFEQ-2: Three-Factor-Eating-Questionnaire, disinhibition, TFEQ-3: Three-Factor-Eating-Questionnaire, hunger.

Figure 4 reports the mediation analysis conducted in individuals with obesity and without regular binge eating. The standardized regression coefficients between the independent general psychopathology and eating behavior variables and EDE-Q score (Path a) were significant for all the independent variables, except for the TFEQ disinhibition and GAD scores (TFEQ cognitive restraint: *p* < *0*.001; TFEQ hunger: *p* < 0.001; BIS: *p* < 0.001; PHQ-9: *p* < 0.001; PSQ: *p* < 0.001) with an explained variance of EDE-Q of R^2^ = 33.6% (*p* < 0.001). The standardized regression coefficient between EDE-Q and admission–discharge BMI difference (path b) was not significant (*p* = 0.071). The bootstrapped indirect effect of the independent general psychopathology and eating behavior variables on admission–discharge BMI difference through the EDE-Q score (path ab) was statistically significant for no scale. Thus, the association between general psychopathological variables/eating behavior and admission–discharge BMI difference was not mediated by the EDE-Q. The direct effect (path c’) was significant between TFEQ disinhibition (*p* = 0.040), BIS (*p* = 0.005) and GAD (*p* = 0.002), but not for TFEQ cognitive restraint, TFEQ hunger, PHQ-9, and PSQ. Standardized regression coefficients and *p*-values for Paths a, c’ (direct), and ab (indirect) are reported in Supplementary Table S3.

## 4. Discussion

Interventions aiming at lifestyle modification represent a relevant element of all treatments for obesity [48]. Treatment guidelines for obesity recommend that individuals with overweight and obesity should be advised to join comprehensive and high-intensity lifestyle interventions, with the supervision of trained staff [49]. Randomized controlled trials (RCTs) support the efficacy of BWLI on weight loss among adults with obesity, which may include a healthy, low-calorie diet, the promotion of physical activity, and structured, behavioral counseling [48]. This is, to the best of our knowledge, the first study to assess a mediation model on the impact of impulsivity, depression, anxiety, stress, and eating behavior to explain weight-loss outcomes in patients with obesity treated with a BWLI.

Our data suggest that general psychopathology and eating behavior may impact weight loss after a BWLI through the mediation of eating disorder pathology. In the overall sample, eating disorder pathology mediated the relationship between weight loss and depression, cognitive restraint, and partially impulsivity (Figure 2). In the subgroup of patients with obesity and regular binge eating, eating disorder pathology mediated the relationship between weight loss and impulsivity, depression, stress, cognitive restraint, and partially disinhibition (Figure 3). In the second subgroup, including individuals without regular binge eating, none of the investigated associations with weight loss were mediated by eating disorder pathology (Figure 4). Thus, regarding our hypotheses, the association with weight loss was mediated regarding most of the investigated variables (besides anxiety) by eating disorder pathology, particularly in patients with regular binge eating.

In more detail, higher impulsivity scores predicted less weight loss. This association was mediated by eating disorder pathology in patients with obesity and regular binge eating, whereas in the overall sample, it was partially mediated, and in the sample without regular binge eating, a direct association was documented. In a recent prospective study, involving patients with obesity receiving no treatment, a conventional weight-loss program, or obesity surgery, high impulsivity has been associated with higher levels of eating disinhibition, which in turn was linked to a higher BMI [17]. Impulsivity, moreover, may have a negative impact for the outcome of a standard BWLI, as documented by another study in patients with obesity [10]. Interestingly, for patients with obesity who underwent an acceptance-based BWLI, which focused on increasing tolerance of negative emotional and physical experiences, the negative effect of impulsivity on the weight outcome was attenuated or even eliminated [10]. Conversely, a more recent study did not find a direct effect of impulsivity on BWLI-associated weight loss [50]. Relevantly, individuals with obesity who have binge eating have been reported to exhibit higher impulsivity scores compared to those without binge eating [18]. Overall, this evidence indicates that individuals with obesity and binge eating may present distinctive impulsivity profiles when compared to those without binge eating, and this relationship may be mediated by eating disorder pathology.

High depressive symptoms negatively predicted weight loss in the overall sample, as well as in patients with comorbid regular binge eating, but there was no direct or indirect association in patients without regular binge eating. Previous studies suggest that comorbid depression may be linked to lower participation rates and less favorable outcomes in BWLI [11,51]. However, these results were not consistently replicated, with one study suggesting that depression should not serve as an exclusion criterion for BWLI [52]. The relationship between depression and weight gain was found to be mediated by pathological eating behavior, specifically emotional eating, in a broad sample of Dutch individuals [20]. Furthermore, BWLI may directly impact depressive symptoms, as documented in an RCT involving patients with obesity and depression [53]. When specifically considering binge eating, atypical depressive symptoms have been linked to higher comorbidity of BED among individuals with obesity [54]. Yet, a recent study has documented that depression scores did not moderate or predict treatment outcomes in patients with obesity and BED receiving cognitive-behavioral treatment or BWLI [55]. Consequently, despite the potential association between depressive symptoms and disordered eating, including binge eating, the evidence in this field remains inconclusive. Depression may influence weight loss through more complex explanatory models, as recently reported by a study identifying depressive symptoms as a mediator between impulsivity, eating disorder scores, and weight loss [37].

In addition to these factors, we explored the relationship between two further psychopathological domains, anxiety, and perceived stress, and the outcome of BWLI. Neither of these factors was found to contribute significantly to the assessed mediation model for the overall sample or for patients without regular binge eating. However, perceived stress was negatively correlated with weight loss, mediated by eating disorder pathology in patients with regular binge eating. Anxiety was directly linked to weight loss in patients without regular binge eating but not associated with weight loss in patients with regular binge eating. Among individuals with obesity, perceived stress may serve as a mediator in the relationship between body image and depressive symptoms [56]. In a recent RCT with patients with obesity treated with a BWLI, post-treatment changes in perceived stress scores showed a positive linear relationship with changes in symptoms of food addiction [57]. Furthermore, a bidirectional association between weight management and stress management is plausible, as stress management has been associated with weight reduction and changes in pathologic eating behaviors in women with obesity receiving a weight-loss program [22]. The evidence for anxiety available in this field is conflicting. In prospective research of individuals with obesity attending a weight-management program, anxiety was associated with lower attendance and completion rates over the follow-up period. However, patients with severe anxiety, when offered additional psychological support, obtained comparable or even better weight-loss outcomes [58]. In a recent study, physical activity appeared to mitigate the effect of anxiety on dysregulated eating behavior in patients receiving BWLI [21]. In summary, the current evidence indicates the need for further longitudinal research to assess the specific domains of eating disorder pathology implied in the relationship between anxiety, stress, and weight outcomes. Such research could pave the way for targeted interventions.

Finally, we investigated the impact of three eating behaviors—cognitive restraint, disinhibition, and hunger—on weight loss. In both the overall sample and the subgroup with regular binge eating, eating disorder pathology mediated the negative relationship between cognitive restraint and weight loss, with no such association in patients without regular binge eating. Disinhibition was directly associated with weight loss in the overall sample and the subgroup without regular binge eating. In the subgroup with regular binge eating, it was partially mediated by eating disorder pathology. Hunger was directly associated with weight loss in the overall sample and the subgroup with regular binge eating, but no association was found in the subgroup without binge eating. Increases in cognitive restraint have been reported as the strongest predictor of weight reduction in a weight loss program for women with overweight/obesity, through the moderating effect of disinhibition [14]. Additionally, cognitive restraint levels have been inversely associated with weight loss in individuals with obesity, mediated by emotional eating and disinhibited eating [23]. Emotional eating serves as a potential predictor for binge eating and, through the mediation of perfectionism, is associated with BMI, as recently reported [59]. We may hypothesize that cognitive restraint, involving the use of cognitive control rather than physiological signals to regulate eating, may alter the habitual response of patients with obesity to food and hunger. Issues related to weight regulation may stem from a conflict between the goals of enjoying food and maintaining weight control (as suggested by goal conflict theory) [60]. Impulsivity has also been found to play a role in the eating disorder pathology of individuals with binge eating and elevated cognitive restraint [61]. More than one component of cognitive restraint, finally, may influence the outcome, as greater weight loss has previously been associated with an increase in “flexible” restraint and a decrease in “rigid” restraint following a weight-loss intervention [62]. The evidence supporting a complex theoretical model of eating disturbances in adult obesity, characterized by direct and indirect effects of cognitive restraint and disinhibition, has been summarized in a recent review [63].

From a global perspective, our results document a predictive model for post-BWLI outcomes, encompassing multiple domains of psychopathology and eating behavior. These domains have been independently reported in different previous studies. For individuals with obesity and regular binge eating, elevated levels of cognitive restraint, disinhibition, impulsivity, depression, and stress may partly account for lower weight-loss outcomes, via increasing eating disorder pathology. A mediation effect for cognitive restraint, impulsivity, and depression was also documented in the overall sample. However, no mediation effects were found in the sample without regular binge eating. We may hypothesize that post-BWLI weight loss represents a complex outcome influenced by the presence of multiple, specific psychopathological traits. These results may have clinical significance since the identification or exclusion of distinct psychopathological domains in a patient who is a candidate for a BWLI could improve the accuracy of prognostic considerations. Even more relevantly, clinical interventions targeting psychopathological traits potentially linked to worse outcomes could improve the effectiveness of BWLI.

This study has both strengths and limitations. All patients included underwent a systematic assessment encompassing various aspects of general psychopathology, eating behavior, and eating disorder pathology. This approach provided the basis for a comprehensive mediation model that integrates diverse variables that have been investigated in separate studies. Mediation models for obese patients with and without regular binge eating were reported, permitting the highlighting of differences in two distinct groups. Finally, longitudinal outcomes for a BWLI are reported, expanding the existing evidence on the effect of these interventions on obesity-related outcomes, a field currently in need of targeted research [64]. Concerning limitations, the assessment of binge eating relied on a single standardized questionnaire, whereas a systematic clinical evaluation of enrolled patients could have allowed for the inclusion of manualized diagnoses of BED. Additionally, the reliability of the PSQ scale in our sample was weak, necessitating caution when interpreting the reported results concerning stress. Since this study presents a per-protocol population, selected after excluding individuals with relevant missing data, severely impaired patients could have been omitted. Lastly, assessing changes in general psychopathological and eating behavior variables between admission and discharge could have expanded our focus on the relationship between these variables, eating disorder pathology, and weight outcomes. Further studies should aim to replicate our results within the context of long-term follow-up and should evaluate the potential impact of BWLI on eventual long-term changes in general psychopathology and eating behavior variables.

## 5. Conclusions

In conclusion, this is the first study to test and present a predictive model for post-BWLI weight loss, encompassing multiple domains of psychopathological and eating behavior, mediated by eating disorder pathology, in patients with obesity with and without regular binge eating. Depression, impulsivity, and cognitive restraint influenced weight loss in patients with obesity, through the mediation of pathological eating behavior. Particularly, one subgroup of patients with obesity who reported regular binge eating showed the most significant and robust associations between weight loss and general psychopathology (depression, impulsivity, stress) as well as eating behavior (cognitive restraint and disinhibition), which were most strongly mediated by eating disorder pathology. In patients without regular binge eating, no mediated associations were observed, but some distinct direct associations (anxiety, impulsivity, and disinhibition) with weight loss have been detected. To personalize the treatment of burdened individuals, these variables, especially eating disorder pathology, should be screened in patients with obesity. Addressing general mental- and eating disorder-related psychopathology may improve weight-loss treatment.

## Figures and Tables

**Table 1:** Main characteristics for the overall sample and the two subgroups.

Variables	Overall Sample (n = 238)	Patients without Regular Binge Eating (n = 139)	Patients with Regular Binge Eating (n = 99)	Group Difference
Females	189 (79.4%)	110 (79.1%)	79 (79.8%)	*p* = 0.901
Age	41.3 +/− 12.1	41.7 +/− 12.2	40.8 +/− 12.0	*p* = 0.575
Previous history of weight loss (yes/no)	83/126 (65.9%)	47/71 (66.2%)	36 (65.5%)	*p* = 0.931
Time Since Maximum Weight Recorded (months)	30.8 +/− 53.5	28.5 +/− 46.0	33.6 +/− 61.6	*p* = 0.102
Maximum previous weight (kg)	129.1 +/− 21.9	128.5 +/− 21.2	130.0 +/− 22.9	*p* = 0.578
Admission weight (kg)	122.6 +/− 19.8	122.2 +/− 19.5	123.1 +/− 20.3	*p* = 0.542
Discharge weight (kg)	120.2 +/− 20.5	119.5 +/− 20.3	121.3 +/− 21.0	*p* = 0.691
Admission BMI (kg/m^2^)	42.4 +/− 5.3	42.3 +/− 5.0	42.5 +/− 5.6	*p* = 0.909
Discharge BMI (kg/m^2^)	41.5 +/− 5.6	41.2 +/− 5.6	41.9 +/− 5.6	*p* = 0.525
Discharge-Admission BMI difference	−0.9 +/− 2.1	−1.1 +/− 2.1	−0.7 +/− 2.1	*p* = 0.258
BIS total score	35.0 +/− 4.9	34.4 +/− 5.2	35.9 +/− 4.4	***p* = 0.037**
GAD total score	7.7 +/− 5.0	6.8 +/− 4.6	9.0 +/− 5.1	***p* = 0.001**
PSQ total score	0.5 +/− 0.2	0.5 +/− 0.2	0.6 +/− 0.2	***p* = 0.001**
PHQ-9 score	9.1 +/− 5.5	8.2 +/− 5.8	10.5 +/− 4.8	***p* < 0.001**
EDE-Q total score	2.9 +/− 1.0	2.7 +/− 0.9	3.2 +/− 1.0	***p* < 0.001**
Binge eating frequency acc. to EDE-Q	6.0 +/− 9.5	0.6 +/− 1.0	13.5 +/− 11.0	***p* < 0.001**
TFEQ cognitive restraint	8.2 +/− 4.0	8.9 +/− 4.1	7.4 +/− 3.7	***p* = 0.009**
TFEQ disinhibition	9.6 +/− 3.8	8.5 +/− 3.6	11.1 +/− 3.6	***p* < 0.001**
TFEQ hunger	9.2 +/− 3.2	6.3 +/− 3.5	7.6 +/− 3.6	***p* < 0.001**

Abbreviations: BIS: Barratt Impulsiveness Scale; BMI: body mass index; EDE-Q: Eating Disorder Examination Questionnaire; GAD: Generalized Anxiety Disorder Scale; PHQ-9: Patient Health Questionnaire-9; PSQ: Perceived Stress Questionnaire; TFEQ: Three-Factor-Eating-Questionnaire. Significant *p*-values are reported in bold.

**Table 2:** Correlations between the considered psychopathological variables.

	BMI Difference	BIS	TFEQ Cognitive Restraint	TFEQ Disinhibition	TFEQ Hunger	GAD	PHQ-9	PSQ	EDE-Q
BMI difference	/	−0.026	0.044	−0.137 *	−0.105	−0.029	−0.107	−0.100	−0.192 **
BIS	/	/	−0.003	0.191 *	0.217 *	0.155 *	0.065	0.086	0.242 **
TFEQ cognitive restraint	/	/	/	−0.281 **	−0.331 **	−0.031	−0.057	0.024	0.164 *
TFEQ disinhibition	/	/	/	/	0.705 **	0.311 **	0.353 *	0.331 *	0.388 **
TFEQ hunger	/	/	/	/	/	0.245 **	0.295 **	0.241 **	0.315 **
GAD	/	/	/	/	/	/	0.712 **	0.748 **	0.435 **
PHQ-9	/	/	/	/	/	/	/	0.660 **	0.505 **
PSQ	/	/	/	/	/	/	/	/	0.476 **

Abbreviations: BIS: Barratt Impulsiveness Scale, total score; BMI: body mass index; EDE-Q: Eating Disorder Examination Questionnaire, total score; GAD: Generalized Anxiety Disorder Scale, total score; PHQ-9: Patient Health Questionnaire-9; PSQ: Perceived Stress Questionnaire, total score; TFEQ: Three-Factor-Eating-Questionnaire. * = *p* < 0.05; ** = *p* < 0.001.

## Data Availability

Data is available by request from the corresponding author.

## Supplementary Materials

The following supporting information can be downloaded at: https://www.mdpi.com/article/10.3390/nu15183915/s1: Supplementary Material Table S1. Mediation model (regression with EDE-Q, direct, and indirect paths) for the overall sample.; Supplementary Material Table S2. Mediation model (regression with EDE-Q, direct, and indirect paths) for patients with obesity and regular binge eating.; Supplementary Material Table S3. Mediation model (regression with EDE-Q, direct, and indirect paths) for patients with obesity and without regular binge eating.
