# Supplementary material for: Mental Health Variables Impact Weight Loss, Especially in Patients with Obesity and Binge Eating: A Mediation Model on the Role of Eating Disorder Pathology
Source: Nutrients. 2023 Sep 9;15(18):3915. doi: 10.3390/nu15183915 (PMC10537364; doi:10.3390/nu15183915)
Supplement: Supplementary file 1 [file nutrients-15-03915-s001.zip › nutrients-2554823-supplementary.pdf]

Supplementary material

Mental Health Variables Impact Weight Loss, especially in Patients with Obesity and Binge Eating: A Mediation Model on The Role of Eating Disorder Pathology

Jacopo Pruccoli, Isabelle Mack*, Bea Klos, Sandra Schild, Andreas Stengel, Stephan Zipfel, Katrin Elisabeth Giel, Kathrin Schag

Table S1. Mediation model (regression with EDE-Q, direct, and indirect paths) for the overall sample.

	**Regressions with EDE-Q (a)**		**Indirect path (ab)**		**Direct path (c′)**	
	**p-value**	**Standardized coefficient**	**p-value**	**Standardized coefficient**	**p-value**	**Standardized coefficient**
**TFEQ cognitive restraint**	**0<.001**	**0.272**	**0.029**	**-0.032**	0.895	0.005
TFEQ disinhibition	**<0.001**	**0.161**	0.058	-0.019	**<0.001**	**-0.185**
TFEQ hunger	**0.046**	**0.073**	0.131	-0.009	**<0.001**	**0.253**
**BIS**	**0<.001**	**0.211**	**0.030**	**-0.025**	**<0.001**	**-0.216**
**PHQ-9**	**0<.001**	**0.360**	**0.032**	**-0.042**	0.136	0.086
PSQ	0.084	0.082	0.173	-0.010	**0.003**	**-0.171**
GAD	0.091	0.083	0.251	-0.010	**0.003**	**0.207**

(a) Standardized coefficients for regressions between psychopathology/eating behavior variables and EDE-Q; (a**b**): standardized coefficients for the indirect path between psychopathology/eating behavior variables and admission-discharge BMI difference via EDE-Q, (**c′**): Standardized coefficients for the direct path between psychopathology/eating behavior variables and admission-discharge BMI difference, controlling for the mediating variable (EDE-Q).

Notes: Variables involved in the final mediation model are reported in bold and underlined. Statistically significant correlations and mediation paths are reported in bold.

Abbreviations: BIS: Barratt Impulsiveness Scale, total score; EDE-Q: Eating Disorder Examination Questionnaire, total score; GAD: Generalized Anxiety Disorder Scale, total score; PHQ-9: Patient Health Questionnaire; PSQ: Perceived Stress Questionnaire, total score; TFEQ: Three-Factor-Eating-Questionnaire.

Table S2**.** Mediation model (regression with EDE-Q, direct, and indirect paths) for patients with obesity and regular binge eating.

	**Regressions with EDE-Q (a)**		**Indirect path (ab)**		**Direct path (c′)**	
	**p-value**	**Standardized coefficient**	**p-value**	**Standardized coefficient**	**p-value**	**Standardized coefficient**
**TFEQ cognitive restraint**	**<0.001**	**0.371**	**0.002**	**-0.075**	0.092	0.085
**TFEQ disinhibition**	**<0.001**	**0.296**	**0.002**	**-0.060**	**0.007**	**-0.140**
TFEQ hunger	.598	0.023	0.621	-0.005	**0<.001**	**0.280**
**BIS**	**<0.001**	**0.185**	**0.005**	**-0.038**	0.984	-0.001
**PHQ-9**	**<0.001**	**0.394**	**0.002**	**-0.080**	0.544	0.047
**PSQ**	**0.005**	**0.146**	**0.044**	**-0.030**	0.328	-0.073
GAD	0.204	-0.069	0.261	0.014	0.979	0.002

(a) Standardized coefficients for regressions between psychopathology/eating behavior variables and EDE-Q; (a**b**): standardized coefficients for the indirect path between psychopathology/eating behavior variables and admission-discharge BMI difference via EDE-Q, (**c′**): Standardized coefficients for the direct path between psychopathology/eating behavior variables and admission-discharge BMI difference, controlling for the mediating variable (EDE-Q).
Notes: Variables involved in the final mediation model are reported in bold and underlined. Statistically significant correlations and mediation paths are reported in bold.
Abbreviations: BIS: Barratt Impulsiveness Scale, total score; EDE-Q: Eating Disorder Examination Questionnaire, total score; GAD: Generalized Anxiety Disorder Scale, total score; PHQ-9: Patient Health Questionnaire; PSQ: Perceived Stress Questionnaire, total score; TFEQ: Three-Factor-Eating-Questionnaire.

Table S3**.** Mediation model (regression with EDE-Q, direct, and indirect paths) for patients with obesity and without regular binge eating.

	**Regressions with EDE-Q (a)**		**Indirect path (ab)**		**Direct path (c′)**	
	**p-value**	**Standardized coefficient**	**p-value**	**Standardized coefficient**	**p-value**	**Standardized coefficient**
TFEQ cognitive restraint	**<0.001**	**0.190**	0.071	-0.014	0.143	0.057
TFEQ disinhibition	0.590	0.023	0.631	-0.002	**0.040**	**0.105**
TFEQ hunger	**<0.001**	**0.147**	0.097	-0.011	0.786	-0.013
BIS	**<0.001**	**0.147**	0.100	-0.011	**0.005**	**-0.107**
PHQ-9	**<0.001**	**0.328**	0.081	-0.025	0.115	-0.103
PSQ	**<0.001**	**0.261**	0.132	-0.019	0.089	-0.098
GAD	0.066	-0.110	0.241	0.008	**0.002**	**0.245**

(a) Standardized coefficients for regressions between psychopathology/eating behavior variables and EDE-Q; (a**b**): standardized coefficients for the indirect path between psychopathology/eating behavior variables and admission-discharge BMI difference via EDE-Q, (**c′**): Standardized coefficients for the direct path between psychopathology/eating behavior variables and admission-discharge BMI difference, controlling for the mediating variable (EDE-Q).
Notes: Variables involved in the final mediation model are reported in bold and underlined. Statistically significant correlations and mediation paths are reported in bold.
Abbreviations: BIS: Barratt Impulsiveness Scale, total score; EDE-Q: Eating Disorder Examination Questionnaire, total score; GAD: Generalized Anxiety Disorder Scale, total score; PHQ-9: Patient Health Questionnaire; PSQ: Perceived Stress Questionnaire, total score; TFEQ: Three-Factor-Eating-Questionnaire.
